# Susceptibility to misinformation as a function of motivated reasoning and news topic: findings from a national representative survey

**DOI:** 10.3389/fpsyg.2026.1942990

**Published:** 2026-10-29

**Authors:** Mauro Bertolotti, Patrizia Catellani, Luciana Fazio, Pierangelo Isernia

**Affiliations:** 1 Department of Psychology, Catholic University of Milan, Milan, Italy; 2 Department of Social, Political and Cognitive Sciences, University of Siena, Siena, Italy

**Keywords:** climate change, conspiracy, fake news, misinformation, Ukraine, vaccination

## Abstract

**Introduction:**

While previous research has shown that susceptibility to misinformation is associated with political orientation and psychosocial processes, the role of misinformation content or topic has so far been scarcely investigated, particularly in research using data collected from representative samples.

**Methods:**

In this study, conducted with a representative sample of Italians (N = 3,025), we investigated the ability to distinguish between real and fake news as a function of political orientation, science-related populism, conspiracy mentality, need for cognition, and need for cognitive closure.

**Results:**

The results showed that, in general, participants who were more susceptible to misinformation held right-wing, populist, and conspiracist worldviews, as well as a low need for cognition and a low need for closure. Regarding misinformation topic, participants tended to perform better on a purely political topic (i.e., the war in Ukraine) than on topics that also had scientific relevance (i.e., climate change, vaccinations).

**Discussion:**

The discussion relates our findings to the broader literature on motivated reasoning and susceptibility to misinformation.

## 1 Introduction

In recent years, there has been a sharp increase in the production and spread of fake news on the Internet, particularly on social media. This phenomenon has attracted the attention of political scientists, who have studied its negative effects on democratic processes and its impact on several high-profile elections worldwide (Chambers, 2021; Jerit and Zhao, 2020), and political psychologists, who have investigated individuals’ susceptibility to misinformation and ways to reduce it (Pennycook and Rand, 2021).

Research has focused on a range of individual variables and processes associated with accepting misinformation as true and sharing it with others (Ecker et al., 2022; Roozenbeek and Van der Linden, 2024). Susceptibility to misinformation has been interpreted as a form of motivated reasoning, as individuals tend to agree with and regard as truthful misinformation that corroborates (and reinforces) deeply held beliefs and worldviews (Peterson and Iyengar, 2021). As such, individual scrutiny and discernment often degrade when news focuses on topics that may embolden (or, conversely, threaten) one’s political party, political identity, or worldview more generally. Further research has highlighted that superficial and inaccurate scrutiny leading to the acceptance of misinformation depends on differences in cognitive style and epistemic motivation, that is, the driving force behind individuals’ information gathering and processing (Uscinski et al., 2024; Douglas et al., 2017), which is not necessarily tied to specific party or group identities, or to the endorsement of certain beliefs and worldviews.

The relative importance and potential interplay of psychological and ideological factors has been the subject of debate (Bordignon, 2023; Scheufele and Krause, 2019). This is particularly relevant for topics that combine political and scientific content. The COVID-19 pandemic (Gabarron et al., 2021) and the ongoing climate crisis (Treen et al., 2020) are cases in which misinformation has been fueled not only by ideological and policy preferences, but also by distrust towards mainstream sources of information, such as public officials (Krange et al., 2021; Merkley, 2020), experts and scientists (Mede and Schäfer, 2020), and flawed representations of scientific consensus.

Our study focused on the psychological and ideological factors associated with the ability to distinguish between true and fake news across different topics. Using a representative sample of Italians, we examined two categories of individual difference factors: those related to epistemic motivations and approaches to knowledge (need for cognition and need for cognitive closure), and those associated with the endorsement of ideologies and worldviews (political orientation, populism, and conspiracy mentality). We examined how these factors were associated with news discernment across three different topics: one with mainly political relevance, specifically the war in Ukraine, and two that combined political and scientific relevance, namely climate change and vaccination.

Besides individual and topic-related factors, how information is presented may also affect judgments of news stories^1^. Inferences drawn from how statements and questions are framed can affect attitudes and opinions, in what has been called the innuendo effect. Wegner et al. (1981) showed that questions and denials in headlines can have negative effects, especially when statements are highly incriminating or sensational. Letourneau and Gawronski (2024) supported these findings, showing that incriminating innuendo can negatively affect attitudes and opinions. Similarly, Kervyn et al. (2012) showed that negative inferences can arise even from positive information when one fundamental dimension of social perception, warmth or competence, is omitted. Pennycook et al. (2020) found that warning labels on headlines can create a truth effect, so that unlabeled false headlines may be seen as valid and contribute to misinformation. Thus, judgments may depend on content and on inferences from how information is communicated.

### 1.1 Individual differences in ideology and worldviews

Past research on political misinformation has shown a link between susceptibility to misinformation and right-wing political orientation (Enders and Smallpage, 2019; Van der Linden et al., 2021), political and social conservatism (Azevedo and Jost, 2021; Hornsey et al., 2018), economic conservatism (Dunlap and McCright, 2011), and right-wing populism (Hameleers et al., 2020). Populism has been found to be closely associated with exposure to and endorsement of misinformation content regarding political and scientific topics. This is because populist narratives often discourage reliance on experts and institutional sources of information, depicting them as corrupt and complicit with elites (Mudde, 2007), encouraging individuals to gather information from alternative sources (Engesser et al., 2017), and to trust common sense (Betz and Johnson, 2004) or their “gut instinct.”

Another phenomenon associated with misinformation is belief in conspiracy theories. Conspiracy theories are narratives and explanations of political or historical events that contradict those commonly accepted by mainstream media and institutional sources and argue for the existence of vast and powerful machinations that control social, political, and economic events to oppress the population or specific social groups (Douglas et al., 2017). False and misleading information is often used as (usually flimsy) evidence for the existence of conspiracies, to engage new audiences in conspiratorial content or reinforce the convictions of believers. In addition to belief in specific conspiracy theories, which can involve both political and historical (Van Prooijen and Douglas, 2017) and scientific themes (Marques et al., 2025; Uscinski and Olivella, 2017), conspiracy mentality has been conceptualized as a predisposition to engage in and endorse conspiracy theories (Bruder et al., 2013; Imhoff et al., 2022). Previous research has shown that individuals with a high conspiracy mentality are more likely to believe fake news and misinformation (Bertolotti and Catellani, 2023; Mancosu et al., 2021; Swami et al., 2017) than those with a low conspiracy mentality.

Given these previous findings on the association between belief in fake news and individual differences in ideology and worldviews, we expected our participants to display different degrees of ability to detect and recognize real and fake news, depending on their political orientation, populism, and conspiracy mentality. Therefore, we formulated the following hypothesis:

*H1*: Right-wing (H1a), populist (H1b), and conspiracist (H1c) worldviews are associated with a lower ability to discriminate between real and fake news.

### 1.2 Individual differences in epistemic motivation

In addition to differences in political views and identity, past research on political and non-political misinformation has found several other social and cognitive factors associated with susceptibility to misinformation (Pennycook and Rand, 2019). Individual differences in information processing, such as dogmatism and cognitive rigidity (Jost, 2017), cognitive reflection (Deppe et al., 2015), and tolerance for ambiguous information (Jost et al., 2009) have been interpreted within the theoretical framework of motivated social cognition (Jost et al., 2003), which posits that information processing and decision-making are guided by epistemic motivations, or needs that direct the acquisition and processing of information (Hennes et al., 2012; Kruglanski, 2013). Two relevant epistemic motivations have been widely investigated in relation to the public understanding of both political and scientific information: the need for cognition (Cacioppo and Petty, 1982) and the need for cognitive closure (Kruglanski and Webster, 1996).

The need for cognition refers to the tendency to engage in and enjoy thinking, as opposed to avoiding cognitively demanding tasks. Individuals with a high need for cognition tend to be interested in learning new things, are comfortable with complex problems, and are willing to change their opinions when presented persuasive messages (Cacioppo and Petty, 1982). Moreover, those with a high need for cognition are more likely to process new information in an accurate and systematic way rather than heuristically. Conversely, individuals with low need for cognition process new information more superficially and with less effort. These individuals are also less likely to notice the factual, causal, or logical inconsistencies that often characterize fake news and are consequently more likely to believe misinformation.

Numerous studies have found a correlational link between a lower need for cognition and a higher susceptibility to misinformation (Leding and Antonio, 2019; Pennycook and Rand, 2019; Salvi et al., 2021). Therefore, we formulated the following hypothesis:

*H2*: A higher need for cognition is associated with a greater ability to discriminate between real and fake news.

The need for cognitive closure is defined as “the desire for a definite answer on some topic, any answer as opposed to confusion and ambiguity” (Kruglanski, 1989, p. 114). This can be a relatively stable personal orientation or can be induced by specific situations such as uncertainty or time constraints. Importantly, individuals with a high need for closure tend to both “seize” available information, being less selective during the information-gathering process than individuals with a low need for closure, and “freeze” it, becoming resistant to change after initial attitudes and opinions are formed based on the first available information. Both characteristics are often displayed by people who are susceptible to misinformation, leading many scholars to identify a high need for cognitive closure as a main risk factor in this phenomenon. However, research on related phenomena, such as the endorsement of conspiracy theories (Albarracín, 2020) and the illusory truth effect (De Keersmaecker et al., 2020), has provided only mixed evidence of an association with the need for cognitive closure. A possible explanation for these mixed findings is that people with a high need for cognitive closure have been shown to be prone to the so-called *fear of invalidity,* that is, the concern that a potential error in judgment may have a high cost (Rietzschel et al., 2007; Thompson et al., 2001). As suggested by Kruglanski and Webster (1996), fear of invalidity can turn seizing into careful pondering, thus increasing the likelihood of more accurate response. In other words, when fear of invalidity is high (through accountability, high stakes, or error salience), people with a high need for cognitive closure expand their information search and outperform those with a low need for cognitive closure on accuracy tasks.

In summary, the urgency to seize and freeze information may make people with a high need for closure more prone to falling for misinformation. On the other hand, the fear of invalidity that may be activated by the task of distinguishing between real and fake news may make these individuals even more careful than people with a low need for closure. Therefore, we formulated the following research question:

Research Question 1 (RQ1) Will a higher need for cognitive closure be associated with a higher or lower ability to discriminate between real and fake news compared to people with a low need for closure?

### 1.3 Similarities and differences between political and scientific misinformation

Political misinformation is a serious concern for the democratic process, and various institutional, educational and communication campaigns have been developed to counter it. Misinformation, however, has spread to public-interest topics that are not merely political but also encompass other domains, such as science. In recent decades, public discourse on climate change (Cook et al., 2015; Moser, 2010) has been severely affected by misrepresentations of scientific consensus (Oreskes, 2004) and false claims (Dunlap and Jacques, 2013), which have slowed climate policy implementation and undermined public support (Lamb et al., 2020). Similarly, misinformation about vaccinations and their alleged harmful effects, particularly on infants, has circulated widely and affected public health in several countries (Jolley and Douglas, 2014).

Research on scientific misinformation has often assumed the existence of a “knowledge gap” on scientific topics, thus directing most efforts towards informational and educational interventions to counter it. More recently, research on misinformation during the Covid-19 pandemic has prompted work that explores the psychological and motivational underpinnings of this type of misinformation (Bertolotti and Catellani, 2023; Van der Linden et al., 2021), adopting approaches more akin to those used to counter political misinformation.

Therefore, it is possible that susceptibility to misinformation is driven by the same cognitive, motivational and social-psychological factors across topics (see Krouwel et al., 2017), but topics that go beyond the strictly political domain pose an additional challenge, since fewer people have access to scientific or medical education and information (Guess et al., 2019). Furthermore, whereas the notions of political propaganda and misinformation are easily accessible and commonly discussed in political discourse (Altay et al., 2024; Sharon and Baram-Tsabari, 2020), people might be less aware of and familiar with scientific misinformation (Cacciatore, 2021; Mede and Schäfer, 2020). For these reasons, distinguishing real news from fake news may be somewhat more difficult when scientific topics are discussed than when mainly political ones are at a stake.

Alternatively, it might be that the relative importance of certain factors in promoting or hindering individual scrutiny of news changes depending on the topic of the news itself. In particular, political preferences and worldviews may disproportionately impact susceptibility to political misinformation but have less impact on topics where the political dimension is less relevant. This would be consistent with the general notion of motivated social cognition (Jost et al., 2003).

In our experiment, we randomly assigned participants to three conditions in which they were exposed to a set of true and fake news headlines about three different topics. We selected the ongoing war in Ukraine as a mainly political topic, because of its relevance at the time of data collection and because the events regarding the Russian invasion and occupation of Ukraine have been surrounded by conspiracy theories and propaganda, making public understanding of the causes and development of the conflict particularly challenging (Hameleers et al., 2024; Shuster et al., 2023). We then chose two further news topics, namely climate change and vaccinations. In the case of vaccination, misinformation and no-vax claims precede the COVID-19 pandemic, although the latter brought renewed attention to vaccine-related misinformation, much of which circulated through social media (Gabarron et al., 2021; Gallegos et al., 2023). Climate-change misinformation has likewise developed within a long-standing and highly politicized public debate (Lewandowsky, 2021). Both topics have been the subject of widespread misinformation and are thus comparable with the case of the war in Ukraine, but they also pertain to domains (climate science and medicine) that go beyond politics and its usual ideological categories. Nevertheless, past research on climate change denial (Biddlestone et al., 2022) and vaccine hesitancy (Gallegos et al., 2023; Ullah et al., 2021), indicates that there is significant overlap between the social groups and political constituencies likely to believe both political and scientific misinformation, and that political campaigns have promoted misinformation on these non-strictly political topics as well (Darius and Urquhart, 2021; Russell, 2023).

As discussed above, we expected that topics with a scientific relevance (i.e., climate change, vaccinations) would pose an additional challenge to participants, compared to a mainly political one (i.e., the war in Ukraine). Therefore, we formulated the following hypothesis:

*H3*: Participants in the climate change and vaccinations news topic conditions will recognize fewer true and fake headlines than participants in the war in Ukraine topic condition.

We also investigated whether the influence of ideology and worldviews differed for each of the three topics included in the survey. These factors might be more influential in the case of political news than on other topics, due to the greater accessibility of political notions and categories when processing information about a clearly political matter than when processing information on less related matters. Some research, however, has shown that ideology and worldviews often shape people’s views also on scientific issues, including climate change (McCright et al., 2016) and vaccinations (Bilewicz and Soral, 2022). Therefore, we formulated an additional research question regarding the possible connections between ideology and worldviews and the three topics we investigated.

Research Question 2 (RQ2). Do individual differences in ideology and worldviews affect the ability to discern between real and fake news on both political (i.e., the war in Ukraine) and scientific topics (i.e., climate change and vaccinations)?

Similarly, we formulated two more research questions regarding the relative impact of epistemic motivations depending on news topics.

Research Question 3 (RQ3). Do individual differences in the need for cognition affect the ability to discern between real and fake news on both political (i.e., the war in Ukraine) and scientific topics (i.e., climate change and vaccinations)?

Research Question 4 (RQ4). Do individual differences in the need for cognitive closure affect the ability to discern between real and fake news on both political (i.e., the war in Ukraine) and scientific topics (i.e., climate change and vaccinations)?

## 2 Methods

### 2.1 Participants

The survey participants were a nationally representative sample (*N* = 3,025) of Italians who joined an opt-in online panel managed by CINT in exchange for a monetary reward. The sample was stratified by gender, age, and area. In the final sample, 51.8% of the participants were female, 47.7% were male, 0.3% were non-binary, and 0.1% did not report their gender. The mean age was 50.7 years (*SD* = 17.3), ranging from 18 to 100 years Regarding educational level, 10.5% had a middle school diploma, 6.9% a professional degree, 47.7% a high school diploma, 11.3% a bachelor’s degree, 17.5% a master’s degree, and 6.1% a master’s or PhD.

### 2.2 Procedure

#### 2.2.1 News stimuli development and pre-testing

Before deploying the survey, we conducted two rounds of pre-testing to select the news headlines to be used as stimuli in the main study. We developed an initial set of real and fake news headlines using actual headlines from Italian online news outlets and fake news spread on social media as a starting point. All headlines were adapted to the same format, consisting of a short title (5–10 words) and a slightly longer text (10–25 words).

In the first pre-test, a sample of volunteers recruited by university students (*N* = 60, age *M* = 28.82, *SD* = 14.08, 65% female, 33.3% male, 1.7% other) were presented with the headlines and asked to indicate whether they believed each headline referred to real or fake news. Each participant was presented with three blocks of six headlines (three real and three fake) on three topics (the war in Ukraine, climate change, and vaccinations), for a total of 18 headlines. The order of the three blocks and the internal order of the six headlines were randomized.

The results showed that the correct classification rate (i.e., real news classified as real and fake news classified as fake) ranged from 57 to 95%, indicating that some headlines were more easily discernible as real or fake than others. We then selected the headlines with a recognition rate between 50 and 75% and revised the others, aiming to obtain a final set of stimuli that were comparable in terms of consensus and difficulty, particularly across different topics. The revised set of 18 headlines was pre-tested with another sample of volunteers (*N* = 22, age *M* = 29.22, *SD* = 11.17, 57.1% male, 42.9% female). Correct classification rates fell within the desired range, and total correct classification scores (*M* = 13.57, *SD* = 3.24 on a 0–18 range) indicated that participants were able to correctly classify an average of 72.2% of the headlines. Therefore, these headlines were selected for the main study.

One example of a fake and true headline for each topic is shown in Figure 1, while the full list of headlines can be found in Supplementary Figure S1.

**Figure 1:**
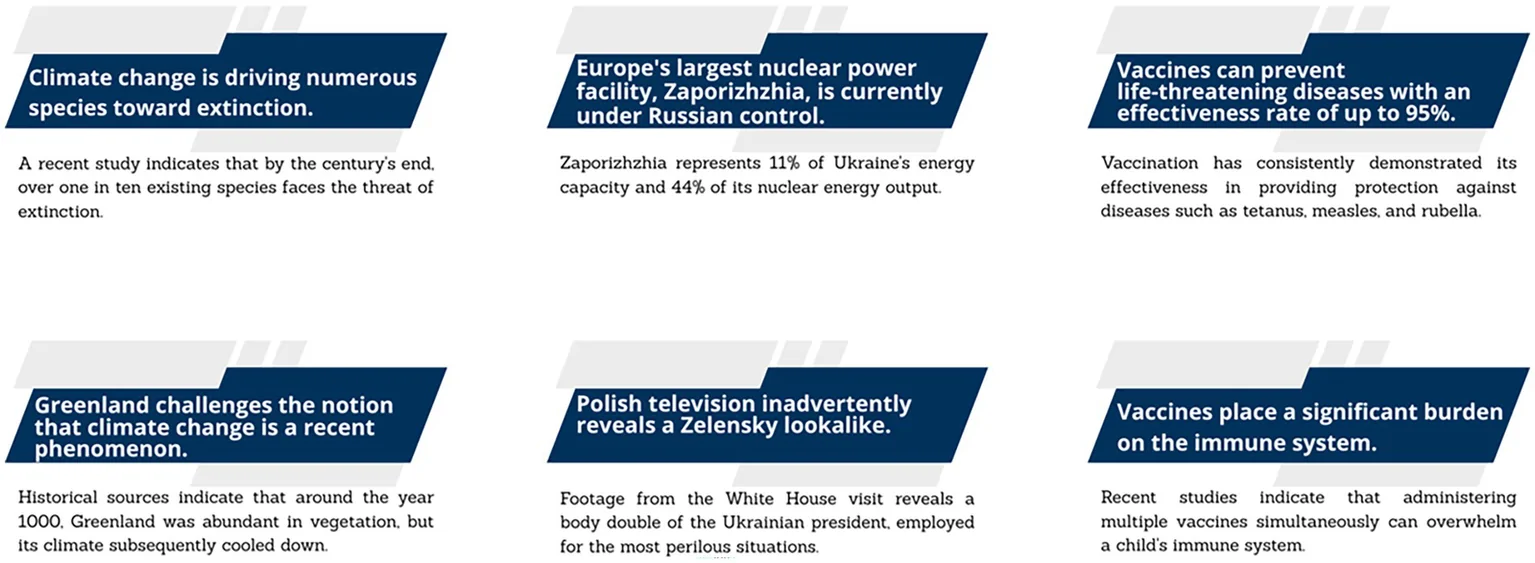
Full text of real (top) and fake (bottom) news headline stimuli used in the experiment.

#### 2.2.2 Questionnaire and fake news recognition task

In the main study, the participants completed a questionnaire divided into two sections. In the first section, we collected data on cognitive dispositions, ideology and worldview measures, and sociodemographic variables (see the Measures section below for more details). In the second section, the participants completed a fake news recognition task adapted from Bertolotti and Catellani (2023). They were randomly assigned to one of three experimental conditions, each associated with a different topic: vaccinations (*N* = 1,003), climate change (*N* = 1,029), or the war in Ukraine (*N* = 993). Participants were then presented with six news headlines, one at a time. After reading each headline, they indicated whether they thought the headline was real or fake news. Participants were then thanked and debriefed.

### 2.3 Measures

#### 2.3.1 Need for cognition

To assess participants’ need for cognition, we used the six-item Italian version of the NFC scale developed by Aquino et al. (2018). Participants rated how representative each statement was representative of themselves on a 7-point scale ranging from 1 (“Not representative at all”) to 7 (“Very representative”). A single average need-for-cognition index was computed.

#### 2.3.2 Need for cognitive closure

To measure the need for cognitive closure (i.e., the desire for epistemic certainty), we used a short version of the scale developed by Pierro et al. (1995), which also consisted of six items. Participants reported their agreement with each statement on a 7-point scale ranging from 1 (“Completely disagree”) to 7 (“Completely agree”).

#### 2.3.3 Conspiracy mentality

To assess conspiratorial attitudes, we used the Conspiracy Mentality Questionnaire (Bruder et al., 2013), which measures participants’ general tendency to believe in conspiracy theories. Participants indicated their agreement with five statements using a scale ranging from 1 (“Completely disagree”) to 7 (“Completely agree”).

#### 2.3.4 Science-related populism

To measure science-related populism, we used a shortened 8-item version of the scale developed by Mede and Schäfer (2020), which explores attitudes toward the “academic elite,” “ordinary people,” and who holds “true knowledge.” Participants reported their agreement with each statement on a scale from 1 (strongly disagree) to 7 (strongly agree).

#### 2.3.5 Political orientation

Participants indicated their political orientation on a 10-point scale, ranging from left (1) to right (10). Unlike previous scales, the response options for this item ranged from 1 to 10, where 1 represented the left and 10 the right. Participants could also choose not to indicate their political orientations. Participants who did so (*N* = 543) were excluded from the analyses using this variable.

#### 2.3.6 News recognition

After reading each headline, participants indicated whether they thought it was “Real” or “Fake.” A single news recognition index was computed, assigning 1 point for fake news correctly recognized as fake and real news correctly recognized as real, and 0 points for incorrectly sorted headlines. The news recognition index ranged from 0–6.^2^

## 3 Results

### 3.1 Preliminary analyses

Table 1 presents the descriptive statistics for the main variables (rescaled to a 0–1 range for comparison) used in the analysis. All indexes showed adequate internal consistency, with Cronbach’s alpha coefficients exceeding 0.68, ensuring the reliability of the measurement instruments.

**Table 1:** Descriptive statistics of the scales and the dependent variable.

Variable	*N*	Mean	SD	Pct (50)	Pct (75)	Min	Max	Cronbach’s Alpha
Political orientation	2,482	0.55	0.20	0.55	0.78	0	1	
Science-rel. populism	3,025	0.51	0.20	0.52	0.64	0	1	0.87
Conspiracy mentality	3,025	0.69	0.21	0.70	0.83	0	1	0.86
Need for cognition	3,025	0.58	0.17	0.58	0.69	0	1	0.68
Need for cog. closure	3,025	0.64	0.18	0.64	0.75	0	1	0.77
Dependent variable	3,025	0.70	0.23	0.67	0.83	0	1	

To check for multicollinearity, Table 2 shows the Pearson correlation matrix, with no correlation higher than 0.5. Conspiracy mentality and science-related populism were moderately positively correlated (*r* = 0.45), indicating some degree of overlap between the two dimensions. Similarly, conspiracy mentality was positively correlated with the need for cognitive closure (*r* = 0.39). In contrast, the correct news identification score, the dependent variable, was negatively associated with conspiracy mentality (*r* = −0.11) and science-related populism (*r* = −0.25), suggesting that greater adherence to these beliefs was linked to poorer performance in identifying truthful news. Political orientation also showed a weak negative correlation with the dependent variable (*r* = −0.22). Finally, need for cognition was not significantly correlated with the other variables.

**Table 2:** Correlation matrix among the main model variables.

	1 Age	2 Education	3 Political orientation	4 Science-related populism	5 Conspiracy mentality	6 Need for cognition	7 Need for cog. closure	8 Dependent variable
1 Age	—							
2 Education	−0.171**	—						
3 Political Orientation	−0.067**	−0.043*	—					
4 Science-related Populism	0.026	−0.086**	0.322**	—				
5 Conspiracy Mentality	0.114**	−0.102**	0.046*	0.455**	—			
6 Need for Cognition	0.065**	0.157**	−0.036	−0.006	0.083**	—		
7 Need for Cog. Closure	0.054*	−0.065**	0.093**	0.331**	0.389**	−0.069**	—	
8 Dependent Variable	0.071**	0.039*	−0.223**	−0.249**	−0.109**	0.057*	0.002	—

*p < 0.05; **p < 0.001; all *N*s = 3,025, except for Political Orientation (*N* = 2,482).

### 3.2 Regression analysis

The model used in this analysis was an OLS regression, treating the dependent variable as a continuous value corresponding to the number of news stories identified correctly.

We conducted two additional analyses (reported in the Supplementary materials) to test the robustness of the results. First, we ran the same model using ordinal logit regression and generalized ordered logit, to account for the ordinal nature of the dependent variable. These alternative ordinal models are presented in Supplementary Tables S1, S2A,B, and show very similar results to the OLS model presented here. Second, we tested the OLS model on two alternative dependent variables based on Signal Detection Theory (SDT), to investigate discrimination accuracy and response bias (Batailler et al., 2022) in participants’ responses. Supplementary Tables S3A,B present the results based on Signal Detection Theory (SDT) and confirm the overall pattern of results. Finally, the model was checked for multicollinearity, which was not a concern, as the main effects VIFs were below 5.

Table 2 reports the results of the regression analysis, showing the main independent variables and the two-way interactions. The results indicated that both the manipulated variable (i.e., the news’ topic) and most of the individual difference variables included in the model had a statistically significant impact on participants’ ability to correctly identify true and false news.

Political orientation performed as expected, as those leaning left were slightly better at selecting the correct answer than those leaning right (Arin et al., 2023), evidenced by the statistically significant negative coefficient, β = −0.155, *p* < 0.001, η^2^_p_ = 0.024, as hypothesized (H1a). The conspiracy mentality index, β = −0.049, *p* = 0.031, η^2^_p_ = 0.002, and science-related populism, β = −0.220 *p* < 0.001, η^2^_p_ = 0.037, also showed significant negative effects, corroborating H1b and H1c. These findings, particularly the larger and more significant coefficient for science-related populism compared to conspiracy mentality and political orientation, suggest that the negative impact of ideology and worldview variables on participants’ ability to distinguish between real and fake news was mainly driven by distrust toward mainstream sources of information (e.g., scientists), rather than specific political beliefs or a general tendency to endorse a conspiratorial worldview.

The results for the epistemic motivation variables were mixed. Need for cognition had the predicted (H2) significant and positive effect on participants’ ability to detect real and fake news, β = 0.068, *p* < 0.001, η^2^_p_ = 0.005, suggesting that individuals who seek greater cognitive certainty and are inclined toward more complex reasoning tend to perform better in the news identification task. This result aligns with previous studies showing that higher levels of this index contribute to individuals being more motivated and thus more effective in detecting and limiting misleading information (Leding and Antonio, 2019; Pehlivanoglu et al., 2021).

Regarding RQ1 on the effect of need for cognitive closure on the ability to distinguish between fake and true news, our analysis showed the presence of a positive and significant relationship (β = 0.099, *p* < 0.001, η^2^_p_ = 0.009). As discussed in the introduction, previous research results left open two possible alternatives regarding this link: greater susceptibility to misinformation among those with a high need for closure, or conversely greater accuracy in these individuals, attributable to a fear of invalidity triggered by the performance demands of the context (Rietzschel et al., 2007; Thompson et al., 2001). Our results clearly point to the second direction. The request to evaluate news headlines and sort them as real or fake likely raised a fear of invalidity among these participants, specifically the concern that a potential error in judgment could incur a high cost (Table 3).

**Table 3:** OLS regression model of participants’ ability to discern real and fake news.

Predictor	95% CI						*p*
	*B*	SE	LL	UL	β	*t*	
(Intercept)	4.260	0.026	4.210	4.310	−0.002		< 0.001
Gender	−0.129	0.051	−0.229	−0.029	−0.048	2.525	0.012
Age	0.006	0.002	0.003	0.009	0.078	3.988	< 0.001
Education	0.011	0.020	−0.028	0.050	0.011	0.544	0.587
Scientific topic 1 (Climate Ch.)	−0.131	0.063	−0.254	−0.007	−0.096	2.074	0.038
Scientific topic 2 (Vaccination)	−0.329	0.063	−0.205	−0.453	−0.241	5.212	< 0.001
Political orientation	−0.078	0.010	−0.098	−0.058	−0.155	7.731	< 0.001
Science-related populism	−0.251	0.026	−0.301	−0.200	−0.220	9.704	< 0.001
Conspiracy mentality	−0.053	0.025	−0.101	−0.005	−0.049	2.154	0.031
Need for cognition	0.091	0.026	0.040	0.142	0.068	3.506	< 0.001
Need for cognitive closure	0.129	0.027	0.075	0.183	0.099	4.701	< 0.001
Sc. Topic 1 X Political. Or.	0.001	0.025	−0.048	0.050	0.002	0.039	0.969
Sc. Topic 2 X Political. Or.	−0.061	0.025	−0.013	−0.110	−0.122	2.467	0.014
Sc. Topic 1 X Sci.-Rel. Populism	−0.041	0.063	−0.164	0.083	−0.036	0.646	0.519
Sc. Topic 2 X Sci.-Rel. Populism	−0.048	0.064	−0.077	0.172	−0.042	0.751	0.453
Sc. Topic 1 X Conspir. Ment.	−0.010	0.061	−0.130	0.109	−0.010	0.171	0.864
Sc. Topic 2 X Conspir. Ment.	−0.201	0.059	−0.084	0.317	−0.185	3.374	< 0.001
Sc. Topic 1 X NF Cognition	0.006	0.063	−0.117	0.130	0.005	0.103	0.918
Sc. Topic 2 X NF Cognition	−0.028	0.062	−0.093	0.149	−0.021	0.448	0.654
Sc. Topic 1 X NF Cog. Closure	−0.005	0.068	−0.138	0.129	−0.003	0.067	0.946
Sc. Topic 2 X NF Cog. Closure	−0.009	0.068	−0.125	0.143	−0.007	0.129	0.898

Model fit: R^2^ = 0.132, F (20, 2,461) = 18.70, *p* < 0.001.

As for the three news topics, we used the Ukraine news condition as the baseline and entered two dummy variables representing the Climate Change and Vaccinations conditions in the OLS model. The results showed that compared to Ukraine, individuals had greater difficulty correctly identifying news related to climate change, β = −0.098, *p* = 0.034, and vaccinations, β = −0.151, *p* = 0.001, omnibus η^2^_p_ = 0.011. When we examined the average scores in the three experimental conditions, we found that selecting the correct answer for Ukraine-related headlines was relatively easier (*M* = 0.73, *SD* = 0.21) than for climate change-related headlines (*M* = 0.70, *SD* = 0.22), and especially for vaccinations-related headlines (*M* = 0.67, *SD* = 0.26). This may be explained by the greater complexity of the latter two topics, as predicted by our hypothesis H3. However, we must also consider the possibility of other influencing factors, such as the relatively high salience of the Ukraine topic at the time of data collection (summer 2024) compared with the other two topics.

To address our research questions regarding the relationships between individual difference variables and the ability to distinguish between true and fake news across different topics, we examined the two-way interactions between each individual difference variable and the three topics included in this study. Both political orientation (as shown in Figure 2) and conspiracy mentality showed strong interactions with the vaccinations condition dummy variable (β = −0.185, *p* < 0.001, η^2^_p_ = 0.003; β = −0.122, *p* = 0.014, η^2^_p_ = 0.006, respectively). This indicates that right-wing and more conspiratorial participants were less able to distinguish between true and fake news about vaccinations than left-wing and less conspiratorial participants. No interaction effects emerged with the epistemic motivation variables (βs < 0.022, ps > 0.653), indicating that their effects were similar across all topics.

**Figure 2:**
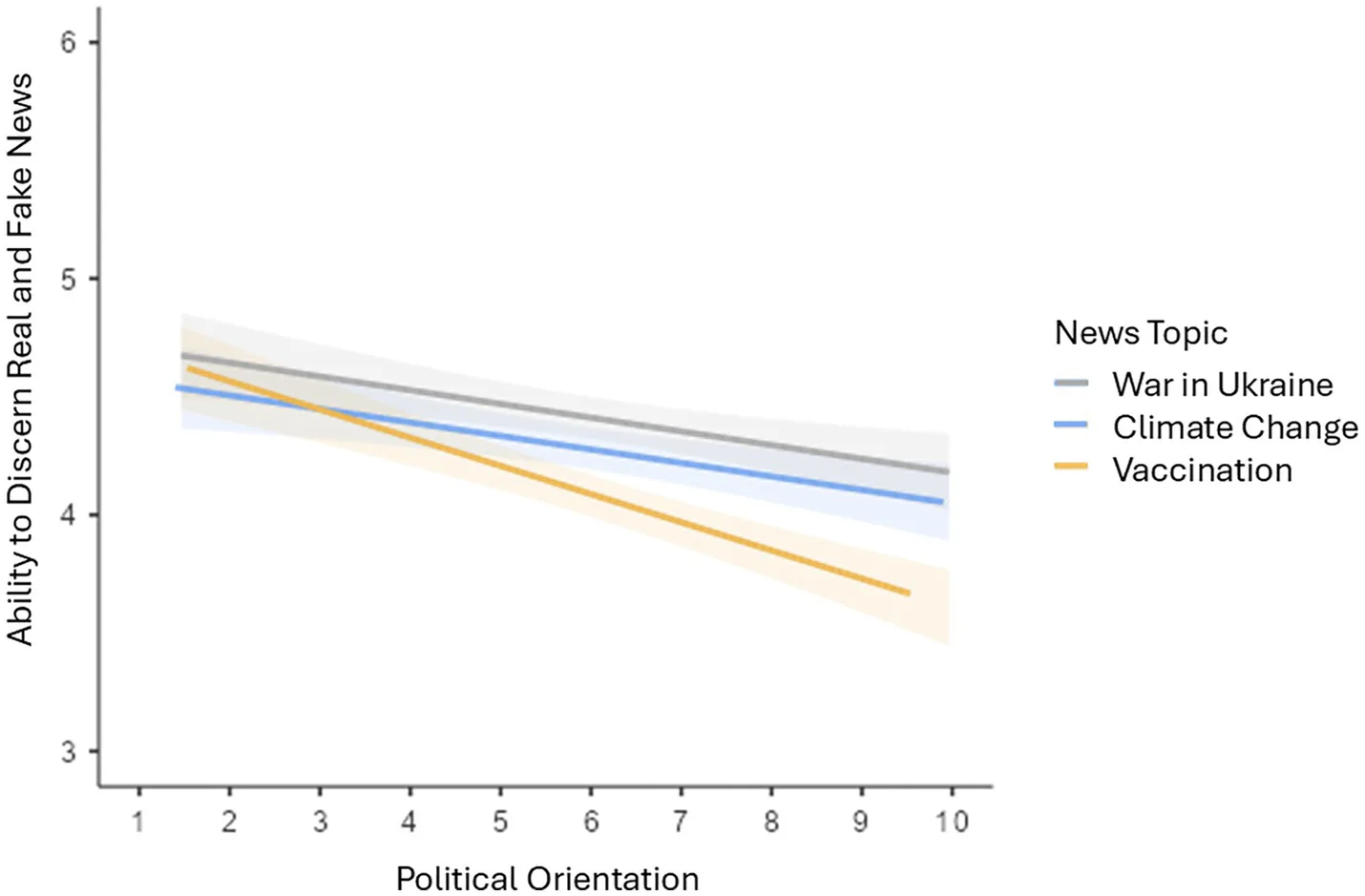
Number of correctly identified headlines as a function of news topic and participants’ political orientation.

## 4 Discussion

Our study results shed light on the relative importance of different predictors of the ability to recognize real and fake news on different topics. Using a headline sorting task within a large, nationally representative survey of the Italian population, we found that right-wing political orientation, science-related populism, and conspiracy mentality were associated with a reduced ability to discern between real and fake news, especially for the topic of, vaccination. We also found that a higher need for cognition and a higher need for cognitive closure predicted a higher ability to discern between real and fake news. Finally, we found that predominantly political headlines were easier to sort into real and fake than headlines on topics that also encompassed the scientific domain. Our results from a representative national sample contribute to deepening our understanding of the predictors of belief in misinformation in several ways.

First, we tested the associations between different psychological predictors of susceptibility to misinformation and participants’ ability to distinguish between real and fake news, as well as the potential interactions between these factors and the news topic. Our findings confirmed our expectations regarding the role of ideological and worldview variables, showing that right-wing political orientation, conspiracy mentality, and science-related populism negatively affect participants’ discernment of real and fake news. Interestingly, we found that the effects of political orientation and conspiracy mentality were particularly strong in one of the two scientific topics we used in our study, namely vaccinations. This may be due to the continued impact of the heated discussions that surrounded the vaccination campaigns during the relatively recent COVID-19 pandemic, which happened to align with existing ideological and cultural lines (Barbieri and Rumore, 2023). Alternatively, this finding might be interpreted in the light of past research indicating that individuals tend to assimilate misinformative content to their ideological views the closer it is to their personal experience (Valecha et al., 2020). It is possible that ideological and conspiracist-motivated reasoning were therefore more apparent on a topic that is more likely to be seen as personally relevant and impactful (vaccinations and their health consequences), than on global issues such as international relations and climate change.

Second, our findings contribute to enriching our understanding of the role of epistemic motivation in making people sensitive to misinformation. A high need for cognition was associated with better performance in recognizing fake and true news. This result substantially replicates previous findings (Leding and Antonio, 2019; Pennycook and Rand, 2019; Salvi et al., 2021), and provides a possible extension to them, showing that the effect of this epistemic variable holds across both political and scientific domains.

Third, our results regarding the link between the need for cognitive closure and susceptibility to misinformation showed a positive, rather than a negative relationship between the two. Although people with a high need for closure often tend to make quick decisions consistent with their worldview, under certain circumstances fear of invalidity can prevail, leading them to reason more accurately. In our study, the task of recognizing true or false news may have primed a motivation for accuracy among those with a high need for closure (Sankaran et al., 2017; Viola et al., 2015) and thus triggered the fear of invalidity that can turn seizing into careful pondering (Kruglanski and Webster, 1996). This interpretation (corroborated also by the alternative analyses reported in the Supplementary Table S3A) suggests that the relationship between the need for cognitive closure and susceptibility to misinformation (De Keersmaecker et al., 2020) is context dependent. If this is the case, debunking and prebunking interventions may benefit from this epistemic motivation by engaging individuals with a high need for cognitive closure in a “quest” to detect fake news in their news feeds or to find real news among the fake.

Finally, we found that news topic does make a difference to the audience’s ability to detect fake news. Participants’ scores in the headline-sorting task were significantly higher when they were presented with real and fake news about the war in Ukraine, and significantly lower when they were presented with news that extended to scientific domains, such as climate change or vaccination. It is possible that the relatively limited public availability of scientific knowledge and information reduced their ability and confidence in distinguishing between real and fake news on topics that required it. Another potential explanation could be participants’ differing levels of concern about fake news on these topics. As mentioned in the introduction, the existence of misinformation is now a well-known and extensively discussed issue in political information (Altay et al., 2024), but this is not the case for scientific misinformation (Cacciatore, 2021; Mede and Schäfer, 2020), and individuals might therefore be less able to detect the stylistic and linguistic signals associated with news on this topic. However, as shown in the Supplementary Table S3B, participants appeared to use an overall more liberal criterion when evaluating scientific news headlines than political news headlines.

### 4.1 Limitations and future research

While this study provides valuable insights into individual behaviors toward scientific and political topics, as well as into the ability to distinguish between true and false news, it has some limitations.

First, the study relied on a cross-sectional survey design, which limited the ability to draw causal inferences. Future research could benefit from longitudinal or experimental designs to examine the effects of prolonged exposure to true and false news, as well as potential changes in behavioral responses and psychological attitudes over time.

Second, future studies should expand and refine the range of topics considered. In particular, the war in Ukraine is a highly salient contemporary political issue that may have facilitated the correct identification of news related to it. Including additional political topics would allow researchers to assess whether misinformation related to scientific topics is more difficult to detect or whether the broader contextual salience of specific events, such as the timing of the survey during the Ukraine war, plays a decisive role.

Third, future research could move beyond text-based news stimuli by incorporating visual elements such as images. Some scholars suggest that images may contribute to the spread of misinformation (Brennen et al., 2021; Hameleers et al., 2020), while also potentially providing cues that facilitate correct identification (Cao et al., 2020). Examining how visual content interacts with individual cognitive and psychological dispositions can further enrich our understanding of misinformation processing.

Fourth, our stimuli, presented information as direct assertions, without manipulating innuendo (Wegner et al., 1981) or measuring participants’ inferences deriving from how the information was presented. T, we cannot determine whether innuendo explains judgments. Although our dependent measure was veracity discrimination rather than evaluative impression formation, the outcome most commonly used in innuendo literature, we cannot exclude that the tone, wording, or framing of our stimuli had an effect on participants’ evaluations. Future research might explore this possibility, for example by contrasting real and fake news with the same informative content, but different phrasing.

Finally, this study focuses exclusively on Italy. Comparative cross-national research would be particularly valuable in assessing whether the observed patterns occur in different contexts.

## 5 Conclusion

Despite these limitations, this study sheds light on the role of psychological dispositions in shaping individuals’ abilities to distinguish between true and fake news. Our findings indicate that right-wing political orientation, conspiracy mentality, and science-related populism are associated with lower probabilities of correctly identifying false information. In contrast, individuals with higher levels of need for cognition and stronger need for cognitive closure perform better on this task. Taken together, these results contribute to the understanding of why certain individuals are more vulnerable to misinformation and offer insights into the types of audiences that fake news is more likely to reach, while also highlighting the types of topics (particularly scientific ones) in which the identification of false information becomes more challenging.

## Data Availability

The raw data supporting the conclusions of this article will be made available by the authors, without undue reservation.

## Footnotes

1 We thank an reviewer for having pointed this issue to us.

2 Our operationalization is a belief-accuracy measure, scored against each headline’s actual veracity status, that should be distinguished from the evaluative-impression measures (e.g., ratings of a target’s honesty or likeability) used in studies of the innuendo effect (e.g., Wegner et al., 1981; Letourneau and Gawronski, 2024), which do not have a ground-truth answer to score against.
