# Supplementary material for: Susceptibility to misinformation as a function of motivated reasoning and news topic: findings from a national representative survey
Source: Front Psychol. 2026 Oct 29;17:1942990. doi: 10.3389/fpsyg.2026.1942990 (PMC13755196; doi:10.3389/fpsyg.2026.1942990)
Supplement: Supplementary file 1 [file Supplementary_file_1.docx]

Supplementary Materials

# 1 Headline Stimuli Used in the Main Study

# Figure S1

# *Full text (translated from the original Italian version) of the news headline stimuli used in the study. For each news topic condition, real news are reported in the respective top row and fake news in the respective bottom row.*

# 2 Robustness Check

Given the nature of the dependent variable (ordinal categorical), we also considered using an ordinal logit regression (ologit/po) model, with the war in Ukraine as the baseline condition for the news topic variable. However, after performing the Brant test (Table S1), we found that the parallelism assumption was violated (Brant, 1990). Therefore, we tested the Generalized Ordered Logit (gologit) model, as it relaxes the proportional odds assumption, that is, the assumption that the effects of the explanatory variables are the same across the dependent variable, which is typical for the ologit model (Williams, 2006; Williams, 2016)^[1]^. Consequently, the gologit model compares successive categories of the dependent variable and allows the effects of the independent variables to vary across them. Thus, different coefficients are obtained for variables that do not pass the Brant test. As shown in Table S2A, the coefficients remain constant for variables that meet this assumption, while they vary for those that do not (Table S2B). Constant coefficients indicate that the effects of the independent variables remain steady across the various levels of the dependent variable (in this case, from zero correctly identified news items to one, from one to two, and so on). Different coefficients indicate that these effects vary across categories. When the coefficients are positive, the probability of being in a higher category of the dependent variable increases. When they are negative, this probability decreases. For the two variables that did not meet the proportional odds assumption, we obtained different coefficients to capture how their effects varied across the categories of the dependent variable. The results for the variable “conspiracy mentality” suggest that a lower level of conspiracy mentality increases the ability to correctly identify news. Likewise, individuals have greater difficulty correctly identifying news related to vaccines (variable “vaccines”) than news about the war in Ukraine. However, this impact is not linear, as its negative effect decreases as the number of correctly identified news items increases, suggesting that this topic has a particularly negative effect on those who have greater difficulty in correctly discriminating information.

**Table S1**

*Brant Test*

| **Variable** | **χ²** | **p-value** | **df** | **Parallel lines violated?** |
| --- | --- | --- | --- | --- |
| All | 155.63 | 0.000 | 100 | Yes |
| Scientific Topic 1 (Climate Ch.) | 7.65 | 0.177 | 5 | No |
| Scientific Topic 2 (Vaccination) | 14.42 | 0.013 | 5 | Yes |
| Political Orientation | 4.71 | 0.452 | 5 | No |
| Sc. Topic 1 X Political. Or. | 1.51 | 0.912 | 5 | No |
| Sc. Topic 2 X Political. Or. | 8.64 | 0.124 | 5 | No |
| Scientific Populism | 5.57 | 0.351 | 5 | No |
| Sc. Topic 1 X Sci. Populism | 8.93 | 0.112 | 5 | No |
| Sc. Topic 2 X Sci. Populism | 5.54 | 0.354 | 5 | No |
| Conspiracy Mentality | 14.68 | 0.012 | 5 | Yes |
| Sc. Topic 1 X Conspir. Ment. | 12.53 | 0.028 | 5 | Yes |
| Sc. Topic 2 X Conspir. Ment. | 8.99 | 0.109 | 5 | No |
| Need for Cognition | 4.51 | 0.478 | 5 | No |
| Sc. Topic 1 X NF Cognition | 3.46 | 0.629 | 5 | No |
| Sc. Topic 2 X NF Cognition | 7.33 | 0.197 | 5 | No |
| Need for Cognitive Closure | 3.28 | 0.657 | 5 | No |
| Sc. Topic 1 X NF Cog. Closure | 2.31 | 0.805 | 5 | No |
| Sc. Topic 2 X NF Cog. Closure | 5.79 | 0.327 | 5 | No |
| Age | 4.66 | 0.459 | 5 | No |
| Education | 2.68 | 0.748 | 5 | No |
| Gender | 4.67 | 0.457 | 5 | No |

**Table S2**

*Gologit model^[2]^*

**A**

| **Variable** | **Coeff. (SE)** |
| --- | --- |
| Scientific Topic 1 (Climate Ch.) | -0.225 (0.514) |
| Political Orientation | -0.785*** (0.228) |
| Sc. Topic 1 X Political. Or. | 0.133 (0.317) |
| Sc. Topic 2 X Political. Or. | -0.634** (0.321) |
| Scientific Populism | -2.125*** (0.393) |
| Sc. Topic 1 X Sci. Populism | -0.384 (0.549) |
| Sc. Topic 2 X Sci. Populism | -0.138 (0.557) |
| Sc. Topic 2 X Conspir. Ment. | -1.287** (0.514) |
| Need for Cognition | 0.772** (0.375) |
| Sc. Topic 1 X NF Cognition | 0.167 (0.536) |
| Sc. Topic 2 X NF Cognition | 0.075 (0.525) |
| Need for Cognitive Closure | 1.098** (0.434) |
| Sc. Topic 1 X NF Cog. Closure | -0.150 (0.583) |
| Sc. Topic 2 X NF Cog. Closure | 0.009 (0.587) |
| Age | 0.699*** (0.178) |
| Education | 0.055 (0.168) |
| Gender | -0.193*** (0.074) |

**B**

| **Variable** | **0** | **1** | **2** | **3** | **4** | **5** |
| --- | --- | --- | --- | --- | --- | --- |
| Scientific Topic 2 (Vaccination) | -0.017 (0.837) | 0.233 (0.596) | 0.487 (0.549) | 0.831 (0.532) | 0.913* (0.527) | 1.016* (0.527) |
| Conspiracy Mentality | -2.869** (1.341) | -0.740 (0.659) | 0.279 (0.478) | 0.413 (0.415) | -0.070 (0.399) | -0.374 (0.419) |
| Sc. Topic 1 X Conspir. Ment. | 1.158  (1.217) | 0.168 (0.665) | 0.103 (0.574) | 0.333 (0.541) | 0.264 (0.531) | 0.058 (0.542) |

LR χ²(35) = 395.91 (*p* < 0.001); Log likelihood = −3873.931; Pseudo *R*² = 0.0486*

*p* < 0.10, ** *p* < 0.05, *** *p* < 0.01.

# 3 Signal Detection Theory (SDT)

Following standard SDT procedures (Batailler et al., 2022), we computed an index for discrimination sensitivity (labeled as d’) and an index for response bias (labeled as c).

Discrimination ability was calculated as d’ = z(H) – z(FA), and response bias as c = -1/2 [z(H) + z(FA)], where z denotes the inverse cumulative distribution function of the standard normal distribution. H refers to the hit rates (i.e., true news articles endorsed as true), and FA refers to the false alarm rates (i.e., fake news classified as true). To avoid infinite values, we applied a log-linear correction (Hautus, 1995), after which z-transformations were used to compute d’ and c. Higher values of d’ indicate greater ability to discriminate true and fake news. Positive values of c reflect a conservative criterion (i.e., a lower tendency to label news as true), while negative values indicate a liberal criterion (i.e., a greater tendency to label the news stories as true regardless of their veracity).

As Table S3A shows, SDT analyses broadly replicate the pattern observed in the main OLS models. Higher levels of need for cognition and cognitive closure are associated with a better ability to identify true and false news stories. Conversely, high levels of scientific populism and right-wing political orientation are associated with lower d’ values, indicating reduced discrimination ability. The effect of conspiracy mentality varies by topic: it is significant only for vaccination, suggesting that people with high conspiracy mentality are less able to correctly identify vaccination-related news stories. Table S3B reports the results for response bias. Scientific populism, conspiracy mentality, and right-wing political orientation are associated with a more liberal response bias, indicating a greater tendency to label news as true regardless of its veracity. In contrast, the results for need for cognition and cognitive closure suggest that their positive association with accuracy (d’) is due to their ability to discriminate, rather than their response strategy (c). Table S3B also shows a topic effect, as the scientific topics (climate change and vaccination) exhibit a more liberal criterion than the political topic (Ukraine). Overall, the SDT analyses confirm the main OLS findings by clarifying the cognitive and decisional processes underlying differences in overall accuracy.

**Table S3A**

*OLS regression predicting d’*

|  | ***B*** | ***p*** |
| --- | --- | --- |
| Climate Change | -0.010 | .971 |
| Vaccination | +0.770 | .006** |
| Need for Cognition | +0.415 | .038* |
| Need for Cognitive Closure | +0.562 | .015* |
| Conspiracy | +0.138 | .491 |
| Scientific Populism | -1.017 | < .001*** |
| Political Orientation | -0.400 | .001** |
| Gender (female) | -0.111 | .005** |
| Education (categories) |  | NS |
| Education: other | -0.515 | .026* |
|  |  |  |
| Political Or. × Climate Change | +0.012 | .945 |
| Political Or × Vaccination | -0.423 | .013* |
| Sc. Populism × Climate Change | -0.153 | .595 |
| Sc. Populism × Vaccination | -0.226 | .437 |
| Conspiracy × Climate Change | -0.099 | .724 |
| Conspiracy × Vaccination | -0.941 | .001** |
| Need for Cognition x Climate Change | -0.60 | 0.83 |
| Need for Cognition x Vaccination | -0.54 | 0.84 |
| Cognitive Closure x Climate Change | 0.17 | 0.95 |
| Cognitive Closure × Vaccination | 0.25 | 0.93 |

*** p < .001, ** p < 0.01, * p < 0.05. Baseline: Ukraine

**Table S3B**

*OLS regression predicting c*

|  | *B* | *p* |  |
| --- | --- | --- | --- |
| Climate Change | -0.346 | .006** |  |
| Vaccination | -0.585 | < .001*** |  |
| Need for Cognition | -0.050 | .583 |  |
| Need for Cognitive Closure | -0.099 | .347 |  |
| Conspiracy | -0.242 | .008** |  |
| Scientific Populism | -0.218 | .021* |  |
| Political Orientation | -0.116 | .038* |  |
| Gender (female) | +0.023 | .199 |  |
| Professional degree | +0.094 | .037* |  |
| High school diploma | +0.063 | .045* |  |
| Bachelor degree | +0.058 | .140 |  |
| Master degree | +0.029 | .411 |  |
| PhD degree | +0.053 | .252 |  |
| Edu other | +0.065 | .542 |  |
| Political Or. × Climate Change | +0.031 | .692 | |
| Political Or. × Vaccination | -0.018 | .813 | |
| Scientific Populism × Climate Change | +0.049 | .707 | |
| Scientific Populism × Vaccination | +0.158 | .236 | |
| Conspiracy × Climate Change | +0.168 | .187 | |
| Conspiracy × Vaccination | +0.148 | .231 | |
| Need for Cognition × Climate Change | -0.089 | .498 | |
| Need for Cognition × Vaccination | +0.158 | .221 | |
| Cognitive Closure × Climate Change | +0.046 | .747 | |
| Cognitive Closure × Vaccination | +0.017 | .908 | |

*** p < .001, ** p < .01, * p < .05. Baseline: Ukraine

1. All analyses were performed using the Stata gologit2 command with the autofit option in STATA19. The autofit option allows you to identify variables that violate the proportional odds assumption and, therefore, those where the proportionality constraints must be relaxed. (Williams, 2006)
2. Coefficients from generalized ordered logit (Gologit). Standard errors in parentheses. To sake of clarity only one set of coefficients is presented for explanatory variables that meet the proportional odds assumption (A): number of news stories 0-1-2-3-4-5 vs six news stories. For the variables that do not meet the assumption (B), six coefficients correspond to every category of the dependent variable: zero news stories, one new story, two new stories, three new stories, four new stories, and five new stories.
